# Correction: Nishikawa et al. Insulin-like Growth Factor-I Reduces Collagen Production by Hepatic Stellate Cells Through Stimulation of Collagen Degradation System via mTOR-Dependent Signaling Pathway. *Biomedicines* 2025, *13*, 566

**DOI:** 10.3390/biomedicines13123095

**Published:** 2025-12-16

**Authors:** Takako Nishikawa, Natsuko Ohtomo, Yukiko Inoue, Mami Takahashi, Hitoshi Ikeda, Kazuhiko Koike, Nobutake Yamamichi, Mitsuhiro Fujishiro, Tomoaki Tomiya

**Affiliations:** 1 Department of Gastroenterology, Graduate School of Medicine, The University of Tokyo, Tokyo 113-8655, Japan; 2 Center for Epidemiology and Preventive Medicine, The University of Tokyo Hospital, Tokyo 113-8655, Japan; 3 Division for Health Service Promotion, The University of Tokyo, Tokyo 113-0033, Japan; 4 Department of Clinical Laboratory Medicine, Graduate School of Medicine, The University of Tokyo, Tokyo 113-8655, Japan; 5 Kanto Central Hospital, Tokyo 158-8531, Japan; 6 Department of Gastroenterology and Hepatology, Saitama Medical University, Saitama 350-0451, Japan; 7 Health Promotion Center, Saitama Medical University, Saitama 350-0451, Japan

**References**

In the original publication [1], citation reference 17 has been retracted; the authors unintentionally cited this retracted reference. The authors have updated the retracted citation reference. The new citation reference appears below. 

17. Kunnumakkara, A.B.; Sung, B.; Ravindran, J.; Diagaradjane, P.; Deorukhkar, A.; Dey, S.; Koca, C.; Yadav, V.R.; Tong, Z.; Gelovani, J.G.; et al. γ-tocotrienol inhibits pancreatic tumors and sensitizes them to gemcitabine treatment by modulating the inflammatory microenvironment. *Cancer Res.* **2010**, *70*, 8695–8705. Erratum in *Cancer Res.* **2018**, *78*, 5181.

**The Zip Codes**

The zip code for [Center for Epidemiology and Preventive Medicine, The University of Tokyo Hospital, Tokyo 113-0033, Japan] in the author affiliations was inadvertently listed as [113-0033]. This should be corrected to [113-8655].

The zip code for [Division for Health Service Promotion, The University of Tokyo, Tokyo 113-8655, Japan] in the author affiliations was inadvertently listed as [113-8655]. This should be corrected to [113-0033].

The authors state that the scientific conclusions are unaffected. This correction was approved by the Academic Editor. The original publication has also been updated.
